# Epidemiological and molecular characterization of Rift Valley fever outbreak in livestock in Burundi, May - November 2022

**DOI:** 10.1371/journal.pntd.0014155

**Published:** 2026-04-10

**Authors:** Canésius Nkundwanayo, Pascal Niyokwizera, Melance Ntunzwenimana, Jean Bosco Ntirandekura, Neilla Ntawuyankira, Annelise Tran, Catherine Cêtre-Sossah, Lionel Nyabongo, John Juma, Max Korir, Reuben Mwangi, Bernard Bett

**Affiliations:** 1 National Veterinary Laboratory, Bujumbura, Burundi; 2 University of Burundi, Bujumbura, Burundi; 3 Institut des Sciences Agronomiques du Burundi (ISABU), Bujumbura, Burundi; 4 Centre de Coopération Internationale en Recherche Agronomique pour le Développement (CIRAD), UMR TETIS, Univ Montpellier, AgroParisTech, CNRS, INRAe, Montpellier, France; 5 CIRAD, UMR ASTRE, INRAe, University of Montpellier, Montpellier, France; 6 International Livestock Research Institute (ILRI), Nairobi, Kenya; Faculty of Science, Ain Shams University (ASU), EGYPT

## Abstract

An outbreak of Rift Valley fever (RVF) was officially reported for the first time in Burundi on 10^th^ May 2022. The outbreak originated in the northern provinces and progressively spread to other regions of the country. This study presents (i) epidemiological investigations that were carried out through a countrywide emergency response and (ii) the characterization of the genotype of the RVF virus that caused the outbreak through phylogenetic analyses. Field teams visited each affected farm, collected data on observed syndromes, species and number of animals affected, farm’s locations, and herd size. Blood, serum and tissue samples were collected from selected clinical cases. Epidemiological data were analyzed using R (version 4.4.2) to determine the spatiotemporal distribution of cases. Mixed effects Poisson regression models were fitted to the data to identify risk factors. A total of 1,739 clinical cases were recorded. Of 100 samples collected and screened using Reverse Transcription Polymerase Chain Reaction (RT-PCR), 36 tested positives. Phylogenetic analyses revealed that the outbreak was caused by an RVFV strain of lineage C, sub-clade C.2.2 of the dominant lineage that was circulating in East Africa, with a close relationship to RVFV that was isolated in Rwanda in 2022. Epidemiological analyses revealed the northeastern region as the epicenter of the outbreak. Multivariable analyses showed that increased RVF cases were significantly associated with high and persistent rainfall and an upsurge in the minimum temperatures that occurred 3–4 months earlier. The analyses conducted provided insights on the risk of RVF in the country. The results would help the development of risk maps and other decision support tools that would be used to manage future risks of the disease.

## Author summary

Rift Valley fever virus (RVFV) is a zoonotic pathogen that causes vector-borne disease in ruminants and humans, severely impacting the health and economy of affected countries. In Burundi, RVFV was reported and officially confirmed for the first time in May 2022. This paper presents a study conducted to genotype the RVFV that caused the outbreak and to identify factors that precipitated the outbreak. Data were generated from field epidemiological investigations in affected areas involving cattle, sheep and goats. Laboratory analysis was conducted to confirm and identify the virus strain, and selected environmental variables were used as predictors based on previous studies that confirmed their association with RVF occurrence. Our study identified an RVFV strain of lineage C, sub-clade C.2.2 as the cause of the outbreak. This is the dominant lineage circulating in East Africa Region, with a closer similarity to the strain isolated in Rwanda in 2022. Environmental factors including rainfall anomalies, minimum temperature, and low slope variance were identified as being significantly associated with increased incidence of RVF cases. The identification of the RVFV strain and environmental risk factors is essential to guide the surveillance efforts in the country and the region.

## Introduction

Rift Valley fever (RVF) is a zoonotic mosquito-borne viral disease caused by the RVF virus (RVFV). The virus was first described in Kenya in 1930 and it is classified into the genus Phlebovirus, family Phenuiviridae and order *Bunyaviridae* [1]. RVFV infection in animals is characterized by fever, abortion, and high mortality rate, especially among young livestock. In humans, RVF is associated with multiple syndromes, a few of which result in death [2].

Re-occurrent epidemics of RVF have been reported in many countries across Africa and the Middle East over the last 10 years. Outbreaks in Senegal, Mali and Mauritania in West Africa; Uganda, Rwanda, Tanzania, Kenya and Sudan in eastern Africa; South Africa and Namibia in southern Africa and the islands of the southwest of the Indian Ocean and the Arabian Peninsula have occurred with substantial public and animal health burden and socioeconomic losses [3]. These outbreaks usually occur following periods of heavy rainfall and flooding that amplify vector populations and increase RVFV transmission [2,4].

RVFV can persist in the environment for long periods between epidemics. Transovarial transmission of the virus among floodwater mosquitoes is believed to contribute to its persistence during interepidemic periods [5]. RVFV is one of the pathogens whose geographical range is gradually expanding due to climate and land use change and increased international livestock trade. An increase in the mean temperature can result in the expansion of the ecological niches of RVFV mosquito vectors. In addition, land use changes such as the construction of dams that support standing water masses have been associated with RVF outbreaks [6]. Animal movement and international trade were associated with the introduction of RVFV to new environments such as the Arabian Peninsula (Saudi Arabia and Yemen) and Madagascar where the first outbreaks officially reported for the first time outside Africa in 2000 [7,8].

Human infection occurs either through direct contact with infected animal tissues, or aerosolized viral particles or indirectly via a bite of an infected mosquito [9,10]. A small proportion of these infections (about 8%) develop into severe hemorrhagic syndrome, with most of the cases manifesting as transient febrile illnesses [11,12]. People whose livelihoods or occupations bring them into direct contact with livestock such as pastoralists, slaughterhouse workers and veterinarians have a high risk of exposure to the disease [13].

On April 10^th^, 2022, the Burundi Animal Health Department reported mass abortions in cattle populations in the north-eastern part of the country. RVFV infections were confirmed by the National Veterinary Laboratory on May 10^th^, 2022 and three teams were set up to train and lead field investigation and follow up from May 18 ^th^, 2022. The Department of Veterinary Services issued an official notification of the RVF outbreak to the World Organization for Animal Health (WOAH) on June 3^rd^, 2022 [14]. The outbreak was first officially reported in Muyinga, Ngozi and Kirundo provinces in the north-eastern parts of the country, and gradually spread southward. Emergency responses were immediately implemented, including field investigations to better define the geographical distribution of the outbreak. Control measures such as animal movement restriction, ban on animal slaughter and milk sales, and community sensitization were also implemented. This paper reports findings from the field veterinary services and laboratory investigations that were carried out to identify factors that precipitated the outbreak. Phylogenetic analyses were also implemented to identify the genotype of the RVFV that caused the outbreak. Our hypothesis was that RVF outbreaks are caused by above-normal precipitation and flooding in ecologies that are conducive for the amplification of RVFV transmission dynamics.

## Methods

### Study area

The Republic of Burundi is a landlocked country in Central East Africa with an area of 27,834 km^2^. It is located between 2°15′- 4°30 S and 28°58′- 30°53′E. The country shares the borders with the Democratic Republic of Congo (DRC) in the West, Rwanda in the North, Tanzania in the East. It is bordered to Lake Tanganyika in the South. The country is divided into eighteen administrative provinces [15,16]. The climate varies with the topography and is characterized by a short rain season from October to December, a long rain season from February to May, a short dry season in January and long dry season from June to September. The annual mean precipitation ranges between 750 mm and 2000 mm, and the annual mean temperature ranges between 14–28°C [15,16]. Agriculture is an important economic activity, employing about 90% of all Burundian’s households. Changes in precipitation directly influence food security and water availability in the country.

### Epidemiological investigations

Outbreak investigations begun in May 2022 following reports from field veterinary services of a sudden increase of abortions and mortalities among livestock. The Burundi Animal Health Department commissioned the investigations. The department has a centralized hierarchical structure led by the Director of Veterinary Services at the national level. The second level is the Provincial Headquarters, managed by the Chief of Animal Health. At the time of the outbreak, Burundi had 18 provinces, but these have since been reduced to five. The Chief of Animal Health oversees several veterinary technicians at the county and zonal levels. The department also engaged community animal health workers at the village level. During the RVF outbreak, thre national rapid response teams were established. Each team included an epidemiologist and a laboratory technician and had two main tasks: (i) training field veterinary staff and (ii) leading investigations in five provinces, namely Kirundo, Ngozi, Muyinga, Kayanza and Cibitoke. An RVF case definition was developed to guide these investigations. A suspected case of RVF was defined as a report within a livestock herd, village or a district of high mortality in neonatal animals, often accompanied by at least one abortion in the herd. Other clinical signs included mucopurulent nasal discharge, or hemorrhagic syndrome in ruminants [2,17,18]. A confirmed RVFV case was from a suspected case that was confirmed as being positive for RVFV based on RT-qPCR test. The field teams used an electronic form administered via smartphones to collect baseline characteristics of herds and individual animals that met the case definition. The form captured data on farm location, number of animals on the farm, number affected, number dead, species, sex and age of animals affected, and their clinical signs.

Field veterinary officers were trained continuously on field investigation procedures and detection of clinical signs of RVF, as experience and knowledge of the disease were limited at the beginning of the outbreak. These trainings were enhanced following the laboratory confirmation of clinical cases. A syndrome surveillance survey based on RVF case definition was established by the Animal Health Department to better report RVF cases and describe the outbreak in space and time at local and national level through initial and follow up reports.

At local level, information (number of cases, species, location) was provided to the county’s veterinary services by the livestock farmers through field regular visits and reported to the head of Animal Health and Production of each province, who was responsible for reporting RVF cases to the national Animal Health Department, on a weekly basis. Data regarding animal population in the affected area were collected from the Animal Production Department and used to calculate RVF incidence.

### Sample collection

A total of 100 cases among those that met the suspected case definition were selected for sampling. For those that had aborted, vaginal swabs and fetal tissues were collected. About 10mL of venous blood was collected via jugular venipuncture. using 18G needles and 10mL syringes; half of this sample was transferred into heparinized vacutainer tubes while the other half was transferred into non-heparinized tubes for serum preparation. Tissue samples collected were stored in 2mL cryovials. Samples were marked using a unique identifier and transported in a cool box to the national veterinary laboratory in Bujumbura for analysis. Samples were stored at −80 °C until analysis. Not all suspected cases were sampled due to cost and logistical considerations.

### Laboratory analysis

#### RVFV genome detection.

Genome detection was carried on the 100 collected samples to detect RVFV RNA. The samples include 82 sera, 6 tissues, 6 tissue swabs, 2 nasal swabs and 4 vaginal swabs that were collected as part of the syndromic surveillance involved suspected cases in goat, sheep and cattle. RNA was extracted from 150 μL of serum samples using QIAamp Viral Mini Kit following the manufacturer’s protocol (Qiagen, Hilden, Germany). Quantitative Reverse Transcription Polymerase Chain Reaction (RT-qPCR) tests were performed using a hydrolyzing probe-based technique (5’ FAM reporter dye and 3’ BHQ1 quencher dye) based on a highly conserved domain located on the L-segment of RVFV [19]. The amplification was performed in a final volume of 15 µL reaction mix containing 7.5 µL KiCqStart® One-Step Probe RT-qPCR ReadyMix™, Low ROX™ (Sigma-Aldrich/MilliporeSigma), 0.75 µL Reverse and Forward primers and probe mix, and 4.75 µL nuclease-free water. A volume of 2 µL of extracted RNA was added to the reaction mix. The reaction was set on an ABI Quant Studio real-time 3 PCR platform (Thermofisher Scientific, Carlsbad, CA, USA) under the following conditions: 50°C for 10 minutes, 95°C for 2 minutes, 45 cycles at 95°C for 3 seconds and 60°C for 30 seconds. The real-time RT-PCR cut-off was set at a cycle threshold (Ct) value of 40 cycles.

### Phylogenetic analyses

#### RVFV targeted amplicon sequencing.

Samples with a cycle threshold (Ct) value less than 30 were selected for sequencing, as that criterion had previously shown higher probability for viral loading and improved sequencing efficiency [20,21].

RNA of selected samples (Ct value < 30) was synthesized to complementary DNA (cDNA) using the Luna Script RT Supermix Kit (New England Biolabs, Hitchin, UK) in a reaction of 20 µL following manufacturers’ instructions. Whole-genome amplification by multiplex PCR was performed using the RVFV primer scheme generated for the two primer pools. A total of 76 primers (38 for each reaction) overlapping the genomic segments of RVFV were used in two separate reactions as already described [21]. Briefly, cDNA was denatured at 95°C for 30 seconds, 95°C for 15 seconds and 63°C for 5 minutes for 35 cycles, and a final hold at 4°C.

#### Generation of consensus sequences.

Consensus sequences for each barcoded sample were obtained from raw sequencing data using an amplicon analysis pipeline (https://github.com/ajodeh-juma/rvfv-amplicon-seq).

In brief, demultiplexed FASTQ data files from the sequencing machine were processed for quality control using FastQC v0.11.9 [22]. Low quality reads and adaptors were trimmed using fastp [23]. For each segment, alignment to the reference genome was performed using bwa-mem [24]. Alignment metrics were obtained using samtools [24] and only samples surpassing a minimum mapping threshold of 500 reads were kept in subsequent downstream analysis. Alignments containing amplicon primers were trimmed using iVar [25]. Using each segment reference (ZH-548 strain) and genome annotation file, variant calling was performed on the primer-trimmed read alignments. Only positions with ≥ 10X genome coverage and with ≥ 20 base quality were used to produce consensus alleles. Regions with lower coverage and base quality as well as those in primer binding regions were masked with N characters. Genome wide, amplicon, amplicon mean and amplicon per base coverages were computed using bedools [26].

### Lineage assignment and phylogenetic analyses

To determine the circulating lineage of RVFV in Burundi, within a regional perspective, we obtained a dataset of the virus genomes from the national center for biotechnology information (NCBI) [27]. We applied the search ‘Rift Valley fever virus AND segment <*segment*> AND <*range*>[SLEN]’, where *segment* was either S, M or L and *range* was the sequence length given as 1521:1690, 3497:3885 and 5764:6404 for small, medium and large segments respectively. As of November 7, 2024, we retrieved a total of 274, 368 and 366 RVF virus genome sequences for small, medium and large segments, respectively. For downstream analyses, we kept sequences that were isolated from the countries in East Africa: Kenya, Uganda, Tanzania, Rwanda, Burundi and Sudan. Other countries in the region that did not have RVFV genome sequences were excluded in the analysis. Together with our generated sequences (>90% coverage), the final dataset following outlier filtering (recombinant, missing metadata) comprised of 108, 154, and 150 sequences for the small, medium and large segments respectively. We obtained the reverse complement of the nucleoprotein (NP) due to the ambisense nature of the small segment. For each dataset (large; n = 150, medium; n = 154, non-structural; n = 108 and nucleoprotein; n = 108), we performed multiple sequence alignment on the coding sequences using MAFFT [28]. We determined the optimal evolutionary substitution models for each dataset using jModelTest [29] and used in the maximum likelihood phylogenetic tree inference using IQ-TREE [30] with 1000 replications in ultrafast bootstrapping procedure. Additional maximum likelihood phylodynamic analysis was performed to estimate the molecular clock and time of the most recent common ancestor of RVF virus in the region. In the heterochronous datasets, we estimated the temporal signal of the sequences by regressing the root-to-tip distances and the time (years). We fitted a migration model in TreeTime to the time calibrated phylogenies to infer the import and export events on the virus in the region. We further performed Bayesian phylogenetic analysis on large and medium segment sequences using Bayesian evolutionary analysis by sampling trees (BEAST) [31] to estimate the time of emergence of the virus in Burundi and the rate of substitution in the virus genomes. We generated time-scaled phylogenies incorporating the uncorrelated log-normal branching and General Time Reversible with Gamma-distributed (GTR + G4) [32] substitution models for the large (n = 150) and medium (n = 154) segment datasets. We ran BEAST with 400 and 300 million Markov chain Monte-Carlo (MCMC) steps, respectively sampling every 40,000^th^ and 30,000^th^ step for large and medium segment sequences. The effective sample sizes (ESSs) were inspected using Tracer [33] for proper convergence to ensure parameters were having > 200 ESSs values. Retrieval and annotation of the maximum clade credibility (MCC) tree was performed using TreeAnnotator with a burn-in of 1001 trees.

### Statistical analysis

The field and laboratory data were merged and exported to R (version 4.4.2) for analysis as a csv file. Descriptive analyses were conducted to determine the distribution of reported cases over time to produce an epidemic curve. Similarly, the distribution of cases was analyzed by province and month, for the period May - November 2022, and mapped out using the *ggplot* () function.

### Risk factor analyses

The risk factor analysis used selected environmental variables (listed in Table 1) as predictors. Their selection was guided by previous causal web models that showed their linkages with RVF occurrence. These variables can be classified into three categories depending on their effects on RVF epidemiology, i.e., (a) the physical factors altitude, slope and soils determine the richness and biodiversity and therefore the disease ecology of an area, (b) human population density and land use/land cover type are proxies for human-environment interactions, and (c) meteorological variables such as rainfall and temperature that influences the timing of climate-sensitive diseases. Human population density is also considered as a proxy for anthropogenic pressure and infrastructure – such as access to markets – that are known to influence infectious disease spread. Choropleth maps of each of the environmental variables were generated and used to explore potential correlations with clinical cases. The maps were generated using the *ggplot* function.

**Table 1:** List of environment variables used as predictors in multivariable analyses to identify risk factors for the Burundi RVF outbreak (May – November 2022).

Name	Spatial resolution	Source	Reference
Rainfall	5 km	CHIRPS	https://data.chc.ucsb.edu/products/CHIRPS-2.0/
Temperature	1 km	MODIS	https://code.earthengine.google.com/
Soil	250 m	ISRIC World Soil Information	https://tinyurl.com/yck4xrrm
Altitude	30 m	Shuttle Radar Topography Mission	https://rcmrd.africageoportal.com/datasets/rcmrd::burundi-srtm-dem-30meters/explore?location=-3.387709%2C29.925250%2C8.43
Slope	30 m	Derived from the altitude data	https://rcmrd.africageoportal.com/datasets/rcmrd::burundi-srtm-dem-30meters/explore?location=-3.387709%2C29.925250%2C8.43
Human population	1 km	WorldPop Hub	https://hub.worldpop.org/geodata/summary?id=139
Land cover	10 m	Sentinel Hub	https://hub.arcgis.com/datasets/rcmrd::burundi-sentinel2-lulc2016/explore

To identify periods of unusual rainfall events, standardized monthly rainfall anomalies were generated, with the reference period being January 1981 – December 2021. Positive values indicated months when there was more rainfall than usual while negative anomalies signified reduced rainfall or drought conditions. Rainfall anomalies were generated in the 5 km raster files before the data were extracted at province level. For each province, mean, minimum and maximum rainfall anomalies were generated for each month. Dummy variables that represented wet (1 for positive anomaly values) or dry (0 for negative values) months were first derived for each of the three rainfall anomaly variables (i.e., mean, minimum and maximum). Subsequently, a rolling number of wet months from 2-4 prior to the time of the outbreak was derived by adding up the dummy variables over time, by province.

Similarly, mean, minimum and maximum monthly temperature estimates were extracted from the temperature raster files (1 km spatial resolution, in ncdf file format) at province level (using the same shapefile format).

Time windows of 2–4 months were defined and minimum or maximum temperature values for a cluster of values for the months in that window was obtained.

The data were analyzed using a Poisson random-intercept regression model. The outcome was the number of cases that was determined in each of the 312 investigations that were done. An additional set of background data generated focusing on the 2020–2021 period when the country did not have active RVF cases. This was offset by the number of livestock in the province where the visit occurred. Data on livestock populations were obtained from the Department of Veterinary Services. The independent variables included mean altitude of the province, mean slope, land use, land cover type and soil types and the various formats of the meteorological variables including mean rainfall, mean minimum and maximum temperature. The province ID was used as the random effect. Univariable analysis was used to screen all the independent variables while the multivariable model included only variables that were significant in the univariable analysis step (p-value = 0.05).

The multivariable model was constructed using the standard backward and forward selection approaches based on the Wald test. The linearity assumption was tested for relevant predictors by fitting quadratic terms in turns in the model.

## Results

The first RVF suspected case in Burundi was confirmed by the National Veterinary Laboratory on May 10^th^, 2022, this prompted the government through its veterinary services department to lead emergency epidemiological investigations.

A total of 312 field investigations were performed between May 18^th^ and November 9^th^, 2022. From these investigations, 1,739 clinical cases were recorded. These included 1,058 cases in cattle, 590 in goats and 91 in sheep. At the same time, 722 fatalities were recorded; cattle comprised the majority (44.7%, n = 473), followed by goats (40.3%, n = 238). Fewer mortalities were observed in sheep (12.1%, n = 11).

### Spatio-temporal distribution of RVF cases

**Fig 1:**
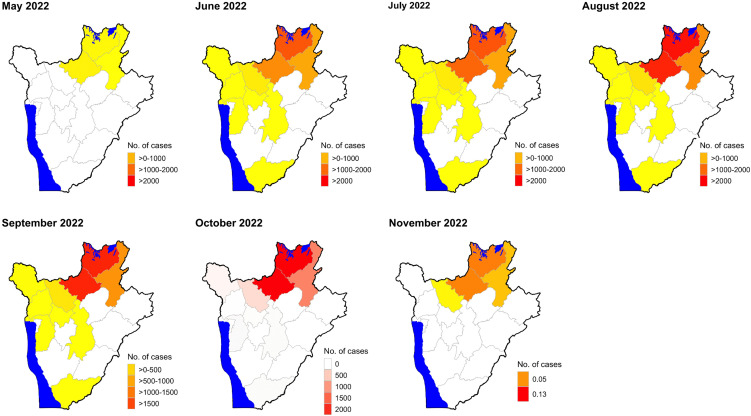
Spatiotemporal distribution of Rift Valley fever (RVF) cases in cattle, goats, and sheep in Burundi, May–November 2022. Burundi shape file used to demarcate the boundaries was obtained from https://www.diva-gis.org/data.html. Administrative boundaries of Burundi were delineated using a shapefile obtained from the DIVA-GIS database.

Fig 1 illustrates the spatiotemporal distribution of RVF cases that were recorded through the field investigations. The outbreak’s epicenter was the northern provinces of Kirundo, Ngozi and Muyinga where most fatalities (n = 576, 79.8%) were observed. RVF cases spread to the rest of the country mainly between June and September 2022, but these receded in the last two months.

**Fig 2:**
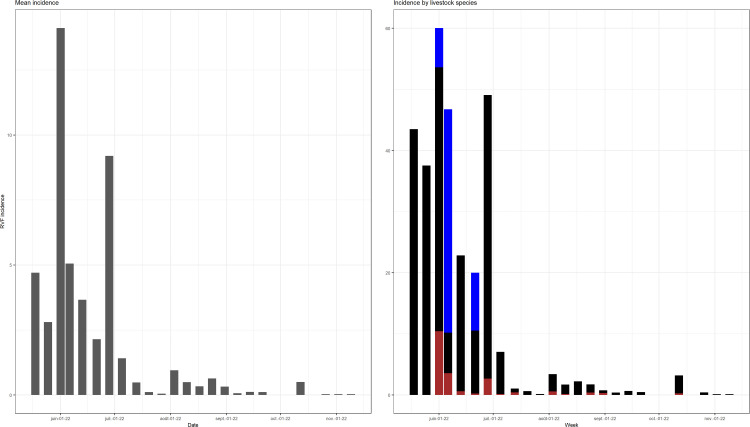
Weekly incidence of Rift Valley fever (RVF) cases by livestock species during the RVF outbreak in Burundi, May–November 2022. Blue, black and brown bars represent sheep, cattle and goats, respectively.

Fig 2 shows the epidemic curve from the outbreak. The highest mean incidence rate was recorded in June (14 for 100000 ruminants) and July (8 for 100000 ruminants) considering 10 000 as the entire population of ruminants. The distribution of the incidence by species indicated that the highest weekly incidence was reported in sheep (60 for 100000 ruminants) followed by cattle (55 for 100000 ruminants) and goats (10 for 100000 ruminants) in June 2022.

### Molecular detection and genome characterization of the RVF virus

Table 2 summarizes results from the molecular analysis of 100 samples collected during RVF outbreak epidemiological investigations.

**Table 2:** RVFV positive genome cases by province and animal species (National Veterinary Laboratory of Bujumbura, Burundi).

Province						Animal species				RVFV RT-PCR positive
Sample type	Kirundo	Muyinga	Ngozi	Kayanza	Cibitoke	Cattle	Goats	Sheep	Total	
Serum	9	16	25	24	8	70	10	2	82	22
Tissue swab	2	4	0	0	0	5	1	0	6	5
Nasal swab	0	1	0	0	1	2	0	0	2	1
Vaginal swab	2	0	1	0	1	4	0	0	4	2
Tissue	0	0	2	3	1	5	0	1	6	6
**Total**	**13**	**21**	**28**	**27**	**11**	**86**	**11**	**3**	**100**	**36**

RVFV genome was detected in 36 of the 100 tested samples (82 sera, 6 tissues, 6 tissue swabs, 2 nasal and 4 vaginal swabs) collected during the field outbreak investigations. All tissues (6/6) and 5 out of 6 tissue swabs were positive for RVF virus as detailed in Table 2.

### RVFV genome sequences

A total of 10 RVFV positive samples were selected (Ct value <30) out of the 36 detected positive RVFV specific RT-PCR samples for genome sequencing using a targeted amplicon approach that utilizes overlapping primers to recover the entire genome of the virus. Generally, there is an inverse relationship between the samples Ct values and the genome coverage rate, indeed we observed higher rates of genome recovery among samples with higher load of virus particles (low Ct values). In total, we recovered 9 RVFV genome sequences, with a coverage ranging from 60% to 95%. Out of the 9 strains, we were able to get 5 yielded near complete genome sequences for each segment with a coverage rate of >90%. RVFV genome sequences (>90%) were submitted to NCBI and given GenBank accessions numbers (Table 3).

**Table 3:** Sequencing and GenBank data with associated metadata of the RVFV strains isolated in different districts, Burundi, 2022. *Sequences not submitted (<90 coverage).

*Strain identification*	*Segment*	*Coverage (rate, %)*	*Accession* *number*	*Location*	*Animal species*	*Isolation Date*	*RVFV RT-PCR (Ct value)*
*BDI0001*	Large	81.54	*	Gikuyo	Cattle	17-05-2022	27
	Medium	87.72	*				
	Small	81.72	*				
*BDI0002*	Large	92.47	OR780625	Ruyaga	Cattle	17-05-2022	19
	Medium	90.42	OR780618				
	Small	93.91	OR780613				
*BDI0003*	Large	97.31	OR780627	Ngozi	Cattle	17-05-2022	19.3
	Medium	91.99	OR780619				
	Small	95.03	OR780614				
*BDI0004*	Large	62.60	*	Ruyaga	Cattle	17-05-2022	25.4
	Medium	84.53	*				
	Small	87.93	*				
*BDI0005*	Large	78.47	*	Kobero	Goat	19-05-2022	30.5
	Medium	91.61	OR780620				
	Small	87.81	*				
*BDI0006*	Large	92.50	OR780627	Kobero	Cattle	19-05-2022	23.4
	Medium	91.74	OR780621				
	Small	92.60	OR780615				
*BDI0007*	Large	92.50	OR780628	Kobero	Cattle	19-05-2022	20.4
	Medium	93.15	OR780622				
	Small	92.60	OR780616				
*BDI0008*	Large	90.88	OR780629	Kobero	Cattle	19-05-2022	19.5
	Medium	91.84	OR780623				
	Small	92.78	OR780617				
*BDI0009*	Large	83.54	*	Ngozi	Cattle	18-05-2022	26.4
	Medium	91.58	OR780624				
	Small	84.32	*				
*BDI0010*	Large	13.68	*	Kobero	Cattle	18-05-2022	26.9
	Medium	25.82	*				
	Small	62.07	*				

### Phylogenetic analysis

**Fig 3:**
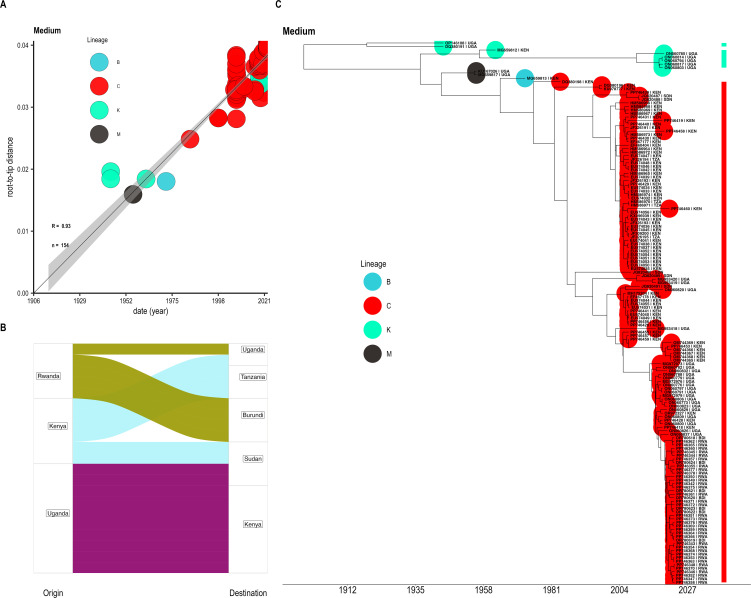
Evolutionary relationships of Rift Valley fever virus (RVFV) strains from the 2022 outbreak in Burundi within the East African regional context. Maximum likelihood phylogenetic tree inference was performed using the medium (M) segment sequences and root-to-tip distance regressed against the collection time in the heterochronous sequences. **(A)** Temporal signal showing sufficient correlation (Correlation coefficient = 0.93) between divergence and time (years) in the dataset. **(B)** Migration model showing the most likely locations for internal nodes in the maximum likelihood tree inferred using TreeTime. **(C)** Time-calibrated maximum clade credibility (MCC) phylogenetic tree was retrieved and annotated from 10,000 trees after discarding 1001 trees as burn-in, here showing the clustering of Burundi, 2022 outbreaks samples alongside different heterochronous sequences from East Africa region. The tips/leaves of the phylogenetic trees are colored according to lineages.

In order to get a complete evolutionary history of the RVF outbreak officially reported in Burundi for the first time in 2022, additional sequences isolated within East Africa were retrieved from NCBI and compared. Given the sufficient temporal signal in the heterochronous sequences (Fig 3A), the migration model fitted to the time-calibrated tree topology showed a close epidemiological link between Rwanda and the Burundi outbreak in 2022 (Fig 3B). We did not observe any differences in the topologies of the maximum likelihood reconstructed trees based on S (Small), M (Medium) and L(Large) segments. Given this observation, we only present results from the M segment. Additional results are presented as supporting information items (S1 Fig to S6 Fig). Bayesian tree inference supported this observation by phylogenetic placement of the circulating strains in Burundi outbreak samples within lineage C cluster of sequences (Fig 3C), specifically within C.2.2, a sub-clade of the dominant lineage in Africa [34]. Bayesian phylogenetic inference showed that the time of occurrence of RVFV in East Africa may have occurred in 1914.58 [95% HPD: 1879.78 – 1940.13]. RVF virus may have first occurred in Burundi in 2019.67 [95% HPD: 2018.48 - 2020.82]. The timing of occurrence of the 2022 RVF outbreak in Burundi coincides with the period when neighbouring Rwanda experienced large-scale RVF cases. The most common recent ancestor of both Burundi and Rwanda are indicated as a cluster of sequences isolated from viruses in Uganda 2018/2019.

### Risk factor analyses

**Fig 4:**
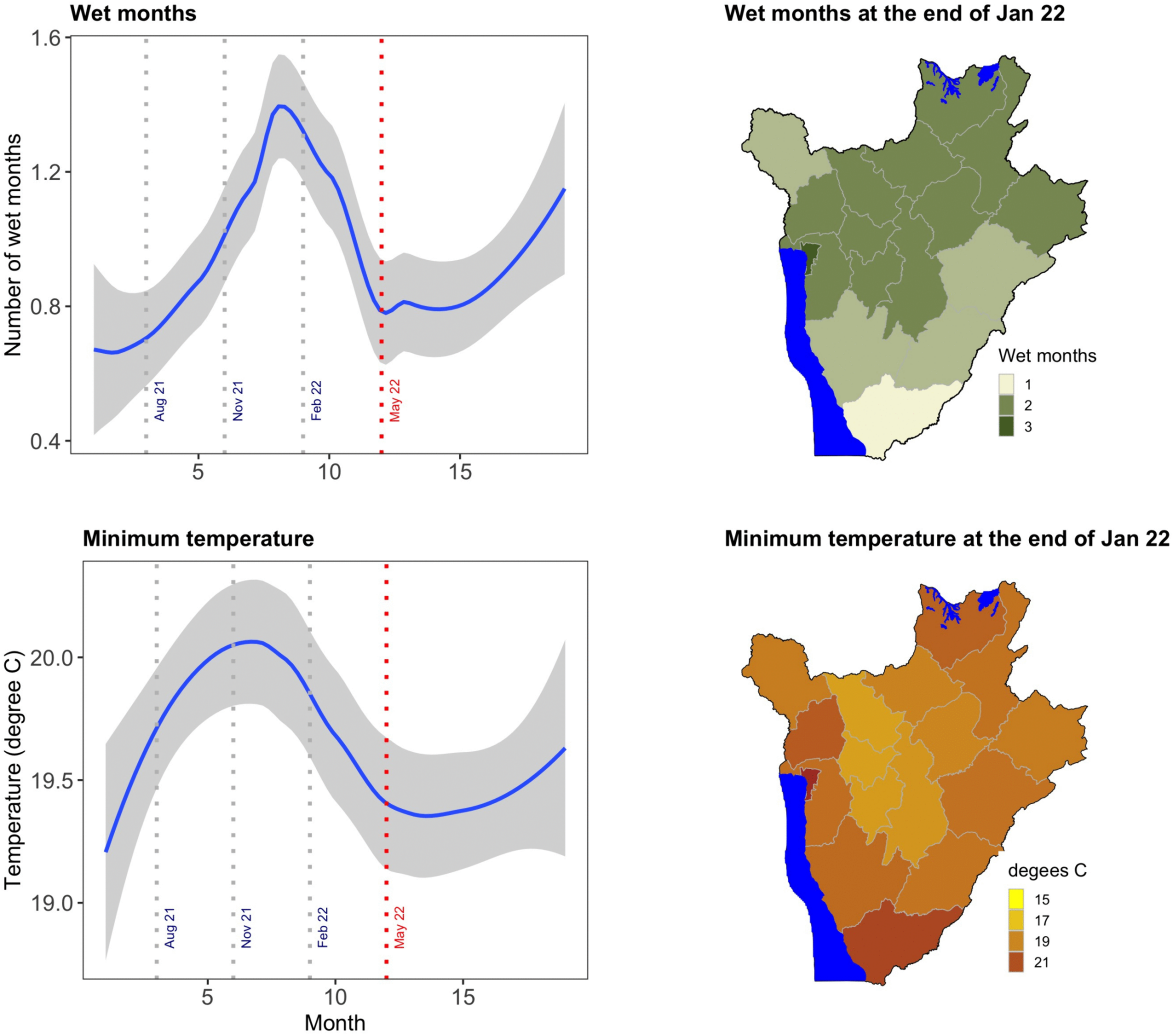
Distribution of meteorological variables used in the study. Temporal distribution of wet months (**top left**) and minimum temperature (**bottom left**) before and after May 2022, the onset of the Rift Valley fever (RVF) outbreak. Maps (**right panels**) show cumulative rainfall (**top right**) and minimum temperature (**bottom right**) at the end of **week 2 (January–February 2022)**. Burundi shape file used to demarcate the boundaries was obtained from https://www.diva-gis.org/data.html.

Fig 4 shows the distribution of the meteorological variables that were used to predict RVF cases. The period that preceded the outbreak -- May 2022 (marked by red dotted vertical line) has been sectioned into quarterly time intervals (Aug – Oct 2021, Nov 2021 – Jan 2022 and Feb – April 2022) to illustrate lag effects of rainfall and temperature. The top-left panel displays the temporal distribution of cumulative wet months between June 2021 and December 2022. The bottom-left panel gives similar patterns for the minimum temperature. In both cases, the cumulative number of wet months and the minimum temperature peaked between November 2021 and February 2022. Maps on the top-right and bottom-right show respectively values of cumulative rainfall and minimum temperatures at the end of January 2022. These values have been selected for display since they provided optimal goodness of fit tests when they were included in both univariable and multivariable models that were used to identify risk factors for the outbreak.

**Fig 5:**
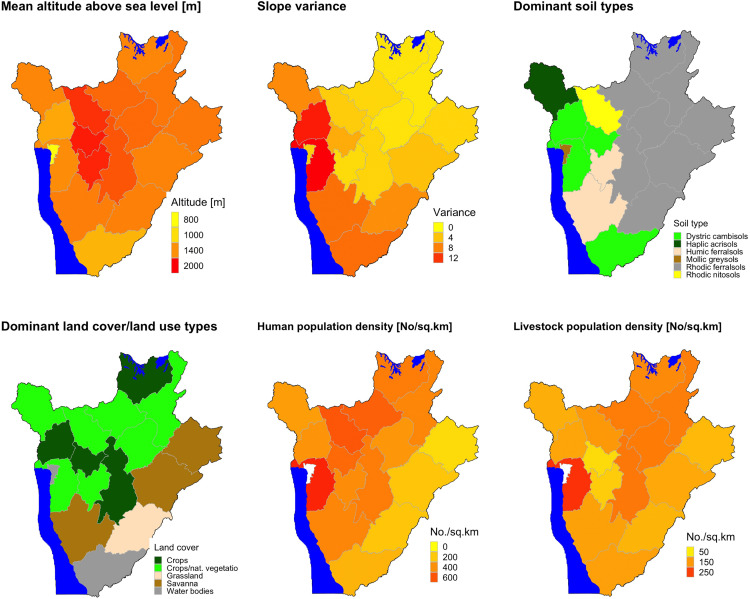
Spatial distribution of environmental and demographic variables in Burundi, 2022.

The distributions of the mean altitude, slope variance, dominant soil type, dominant land use/land cover type (by province), human and livestock population densities across the country are given in Fig 5. These data show that the northern region that was greatly affected by the outbreak is a highland with a gentle terrain (with a gentle slope). The region also has rhodic ferral sols as the dominant soil type. The lower panel of the figure shows that crop farming as well as livestock husbandry are the main livelihood activities. Moreover, the region has very high human and livestock population densities.

Maps show mean altitude, slope variance, dominant soil types, dominant land cover types, and human and livestock population densities. Burundi shape file used to demarcate the boundaries was obtained from https://www.diva-gis.org/data.html.

Tables 4 and 5 provide results from univariable and multivariable random effects statistical models used to identify risk factors for the RVF outbreak. Three variables were found to be significant in all analyses: the cumulative number of wet months, the minimal temperature and the slope variance. The random effect (province ID) was also significant, as its profile likelihood confidence interval (CI) did not include a zero. The cumulative number of wet months failed to meet the linearity assumption and was therefore used in the model as a categorical variable. Both minimum temperature and the cumulative number of wet months were lagged by three months.

**Table 4:** Results of the univariable analysis used to assess the association between selected independent variables and the number of RVF cases in livestock in Burundi (May – November 2022).

Variable	Levels	n	Parameter estimate	
			Point estimate	95% CI
Soil type	Haplic acrisols	26	1.37	-1.40, 4.16
	Rhodic ferralsols	104	2.86	1.08, 4.63
	Rhodic nitisols	26	2.38	-0.37, 5.15
	Dystric cambisols	104	0.00	–
Dominant land use/land cover type	Cropland	104	-1.39	-3.62, 0.80
	Water bodies	26	-2.41	-6.03, 1.22
	Crop land + natural vegetation	130	0.00	–
Human population ^a^	–	–	0.32	-0.84, 1.50
Mean altitude ^a^	–	–	0.25	-0.93, 1.44
Maximum altitude ^a^	–	–	-1.19	-2.04, -0.34
Mean slope	–	–	0.60	-0.52, 1.71
Slope variance	–	–	-0.28	-0.48, -0.07
Minimum temperature ^b^	3899	–	3.09	3.08, 3.72
Cumulative wet months ^c^	2483		1.93	1.81, 2.05

^a^Factor fitted as a scaled variable to reduce the model eigenvalue

^b^Minimum temperature lagged by 3 months. It gave the least AIC estimates compared to other formats of the temperature variable (i.e., mean and maximum temperature in 3 months lagged by 3 months and mean, minimum and maximum temperature in four months lagged by 3 months).

**Table 5:** Outputs of a random effects Poisson regression model used to identify risk factors for clinical RVF infections in livestock in Burundi (May – November 2022).

Variable	Level	Parameter estimate	
		Point estimate	95% confidence interval
Intercept	–	-103.15	-115.53, -91.82
Minimum temperature	–	4.82	4.25, 5.42
Number of wet months	1	0.79	0.47, 1.11
	2 -3	3.26	3.02, 3.52
	0	0.00	–
Slope variance	–	-0.81	-1.46, -0.17

Log likelihood -902.3.

When controlling for other two significant variables, a rise in minimum temperature was significantly associated with an increased RVF cases. Similarly, more wet months were associated with increased number of cases. The increase was particularly marked when wet months rose from 1 to 2 or 3, compared to an increase from 0 to 1. Lastly, areas with low slope variance had more cases than those with variable slopes.

#### Univariable and multivariable analysis

## Discussion

The first laboratory-confirmed case of RVF in Burundi was on May 10^th^, 2022. The country was affected at a time when other countries in the region, particularly Rwanda in the north, was experiencing a similar sanitary health problem [35]. The outbreak was first officially reported in Ngozi, Kirundo and Muyinga provinces in the north, then the disease progressively spread southward to other provinces including Kayanza, Cibitoke, Bujumbura Rural, Karusi, Gitega, Mwaro, Makamba and Rumonge. Given the prolonged time interval between alert and laboratory confirmation, the animal movement measures were not fully implemented. Also, the distance between animal markets from one province to another is relatively short. Therefore, the delay may have contributed to the spreading of the disease. Cattle were the most affected livestock species compared to goats and sheep, which is contrary to what is usually known: that sheep and goats are more susceptible to the disease than cattle [36,37]. The small number of sheep in Burundi compared to goats and cattle might have impacted the result of the study. Second, the socio-economic consideration of cattle is likely more important than that of sheep and goats, and this could have led to under-reporting of cases in small ruminants. The country suffered heavy losses from livestock morbidity and mortality, trade embargoes on live animals and livestock products and expenditures on emergency responses [14]. The outbreak also occurred at a time when the country was yet to recover from the COVID-19 pandemic [38].

This study was designed to characterize the outbreak through (a) genomic and phylogenetic analysis of the RVF virus to determine its relationship with regional isolates, and (b) identify environmental factors that precipitated the outbreak. For the first question, whole genome sequences of the virus were obtained from segment M. Phylogenetic analyses showed clustering with lineage C strains from East Africa, confirming that this lineage is the main variant in the region. Within the clade C, the Burundi isolates form a sub-cluster C.2.2 with sequences from Rwanda with ancestral sequences pointing to 2018/2019 Uganda isolates. Our Bayesian time-calibrated phylogeny showed that the virus responsible for the 2022 outbreak was likely introduced in the country in 2019 [95% HPD: 2018.48 – 2020.82]. This coincides with the period when Rwanda experienced its first large-scale outbreak following heavy rainfall [34]. The availability of these genomic data is important for RVFV surveillance in the region. This study highlights the utility of molecular epidemiology and surveillance of this mosquito-borne virus in monitoring circulating lineages following outbreaks in regions previously considered non-endemic.

The epidemiological analysis identified rainfall anomalies and minimum temperature, lagged by three months, and low slope variance as being significantly associated with increased incidence of RVF cases. The co-occurrence of these environmental factors and the presence of susceptible hosts may have contributed to the attainment of minimal criteria for RVF outbreaks. Meteorological factors (rainfall and temperature) represent dynamic processes that determine critical conditions for the development and amplification of RVF virus’s vector populations, while slope is a static factor that influences the susceptibility of an ecology to RVF directly or indirectly by determining rate of water accumulation for mosquito colonization and breeding. Previous analyses have used normalized difference vegetation index (NDVI) to represent dynamic ecological changes that precede RVF outbreaks [39]. However, NDVI is an intervening variable in the RVF causation pathway. This approach is likely to introduce bias by masking the effect of key predictors. NDVI was excluded as it likely represents an intervening variable in the causal pathway linking climatic factors to transmission of RVFV, and its inclusion could introduce overadjustment bias. However, alternative modelling frameworks may offer complementary insights.

Rainfall helps create mosquito breeding sites by filling up natural and man-made depressions, crevices and containers with water. Unlike other mosquito-borne diseases, RVF outbreaks often requires above normal and persistent rainfall events to enable the breeding of large populations of mosquito vectors probably because these outbreaks require a much higher thresholds for occurrence than those for the other arboviruses. This assumption (for the high threshold for RVF transmission) is partly informed by observations from sero surveillance studies in RVF endemic sites where mosquito sampling and screening often report a wide spectrum of arboviruses of public health importance, yet RVF virus is infrequently detected [40,41]. While there is not any information on the range of vectors for RVF in Burundi, it is generally believed that flooding should be sustained for about 5–7 weeks of a “functional cluster” of mosquito species that comprise the primary vectors which seed infection in a population of susceptible animals, and secondary vectors that amplify the transmission processes [42,43]. An increase in temperature over the physiological limits of mosquitoes increases the development rates of aquatic stages. It also increases the feeding intervals of mosquitoes therefore hastening the vector host contact [44,45].

Several other environmental variables were not significant in the analysis, yet they may have important roles in RVF occurrence. The affected region had high human and livestock population densities which may be important for enhancing pathogen-vector-host interactions. While human cases were not reported in this outbreak to our knowledge, the high population density increased the risk of human infection. These observations indicate an urgent need to develop public and animal health delivery systems across the country to improve the existing surveillance systems.

The extreme rainfall and temperature events and a higher proportion of RVF cases were detected in the northern parts of the country although moderate rainfall and additional clinical events of the disease were detected in many other parts of the country. The occurrence of the disease in areas that did not experience extreme climate events could be explained in two ways. First, it is likely that RVF is endemic in the country and the enhanced surveillance during the outbreak period might have enabled greater vigilance that led to the detection of multiple clinical events across the country. Second, livestock movement might have aided the dissemination of the virus particularly in the earlier periods of the outbreak when quarantine measures had not been enforced.

This study used a multidisciplinary approach to complete both the sequencing and phylogenetic analysis of the virus and epidemiological analysis of the outbreak. Both approaches utilized the state-of-the-art methodologies to achieve an in-depth characterization of the outbreak. The methods used for the whole genome sequencing were developed recently and they have been applied to analyze RVF virus isolates from Kenya [21] and Rwanda [34]. Similarly, the epidemiological analyses began with an elaborate pre-processing of the predictor variables to determine the number of wet months and the minimum temperature values that would be informative. Raw meteorological station data of these variables (rainfall and temperature) were fitted in preliminary versions of the model, but they did not provide significant results. This highlights that effective RVF prediction is achievable by pre-processing predictor variables to extract signals that best capture underlying patterns. This includes capturing the lag effects, mainly involving the dynamic variables but in some cases spatial lag effects occur.

The statistical model developed from this study can be used reliably to predict RVF in the country. Similar models have been developed in the past, notably the statistical model that was proposed by Anyamba et al. (2009). We believe that our model provides a better prediction tool since it combines physical (e.g., slope) and climatological (rainfall and temperature) variables that are critical for RVF occurrence, yet they have not been explicitly captured in the published models. It is also critical to point out that models that include proximal variables (such as rainfall and temperature) on RVF prediction can perform better than those that include distal or intervening variables such as NDVI for they are likely to afford a longer lead time.

The study had a few weaknesses. The key limitations are related to syndromic case classification, incomplete laboratory confirmation, absence of entomological and human data, possible undetected RVF cases prior to circulation, potential surveillance bias, and absence of sequence data from some of the neighbouring countries, which may affect inferences on the transmission dynamics of Rift Valley fever virus.

First, not all the suspected cases captured in the database were confirmed using laboratory screening tests due to cost and logistical considerations.

Most of them were recorded as RVF syndromes based on the RVF case definition. While this may introduce misclassification bias of the outcome, the bias is not expected to be substantial since the syndromes had similar signs as the cases that were laboratory confirmed by RT-PCR and occurred in the same locations. Secondly, the timing of the outbreak was linked to the period of time when confirmatory tests were done. The outbreak is suspected to have started in April 2022. This implies that the effective lag effects of rainfall and temperature could be about 1 month shorter that has been reported in the paper. This calls for the need to sensitize surveillance unit in the country with respect to identifying and recording dates of disease outbreaks more accurately. Third, only livestock cases were captured in the surveys conducted. The surveillance team did not report any human cases. More work is needed to build capacity on the detection of human RVF cases in line with the International Health Regulations of 2005 (https://www.who.int/publications/i/item/9789241580496). This should also focus on implementing measures to prevent and control the transmission of RVFV to humans. Similarly, no data on vectors were collected. Vector surveys should have been undertaken to identify the vector species present and potentially involved in the epizootic. There is a need to invest in capacity building for routine vector surveillance and control to better anticipate and prepared for RVF emergence. The last key limitation of the study is the uneven regional genomic sampling and the absence of sequence data from some neighbouring countries, which may have helped improve understanding of the phylogeographic patterns of RVFV circulation. Future efforts should prioritize strengthening cross-border collaborations, data-sharing with neighbouring countries, and capacity building in genomic surveillance for a better understanding of transboundary transmission dynamics of RVFV.

## Conclusion

This study generated the first batch of whole genome sequences of the RVFV from livestock in Burundi and enabled analysis of its ancestral origin and circulating genetic lineages. The results showed that a single RVFV lineage C circulated during the 2022 outbreak, and is the dominant variant in the region The availability of these genomic data is important for RVFV surveillance efforts in the East-Africa region. The study identified the environmental factors associated with RVF cases. There is a need for RVFV vector evaluation to further improve RVF risk mapping in Burundi. The findings of this study highlighted the need for efficient national and regional collaboration in order to control this emerging transboundary zoonotic disease.

## Supporting information

S1 Data Rift_Valley_Fever_Laboratory_Samples_results_from_5_provinces. (XLSX)

S2 Data Timely_Rift_Valley_Fever_cases_report. (CSV)

S3 Data AppGenBank_ accession_ numbers_ for _nucleotide_ sequences. (DOCX)

S1 Fig RVFV_Large_maximum_clade_credibility_tree. (TIF)

S2 Fig RVFV_Large_beast. (TIF)

S3 Fig RVFV_Large_reetime. (TIF)

S4 Fig RVF_Medium_beast. (TIF)

S5 Fig RVFV_Large_Medium_maximum_clade_credibility_tree. (TIF)

S6 Fig RVFV_phylo_mltrees. (TIF)
