# Supplementary material for: Epidemiological and molecular characterization of Rift Valley fever outbreak in livestock in Burundi, May - November 2022
Source: PLoS Negl Trop Dis. 2026 Apr 10;20(4):e0014155. doi: 10.1371/journal.pntd.0014155 (PMC13068329; doi:10.1371/journal.pntd.0014155)
Supplement: S3 Data — (DOCX) [file pntd.0014155.s003.docx]

**GenBank accession numbers for nucleotide sequences**

BankIt2760903 BDI0002    OR780613
BankIt2760903 BDI0003    OR780614
BankIt2760903 BDI0006    OR780615
BankIt2760903 BDI0007    OR780616
BankIt2760903 BDI0008    OR780617
BankIt2761051 BDI0002    OR780618
BankIt2761051 BDI0003    OR780619
BankIt2761051 BDI0005    OR780620
BankIt2761051 BDI0006    OR780621
BankIt2761051 BDI0007    OR780622
BankIt2761051 BDI0008    OR780623
BankIt2761051 BDI0009    OR780624
BankIt2761151 BDI0002    OR780625
BankIt2761151 BDI0003    OR780626
BankIt2761151 BDI0006    OR780627
BankIt2761151 BDI0007    OR780628
BankIt2761151 BDI0008    OR780629
